# Patient and Physician Perceptions of Prostate-Specific Antigen Testing Among Black Individuals

**DOI:** 10.1001/jamanetworkopen.2025.30946

**Published:** 2025-09-08

**Authors:** Jenney R. Lee, Dante’ Morehead, Ben Young, Victor Tolbert, John Masembe, Garrett Britt, Lars Neuenschwander, Kyle Schuppe, Richard Pelman, Debi Johnson, Vida Henderson, Burcu F. Darst, Patricia Egwuatu, Sung Min Kim, Erika M. Wolff, John L. Gore, Yaw A. Nyame

**Affiliations:** 1 Department of Urology, Center for Health Outcomes Research and Dissemination, University of Washington, Seattle; 2 Black and African-Descent Collaborative for Prostate Cancer Action, Seattle, Washington; 3 Washington State University, Spokane; 4 Washington State Urology Society, Mill Creek; 5 Public Health Sciences, Fred Hutchinson Cancer Center, Seattle, Washington; 6 Kaiser Permanente, Seattle, Washington

## Abstract

**Importance:**

Black individuals have a twofold higher rate of prostate cancer death in the US compared with the average population with prostate cancer. Few guidelines support race-conscious screening practices among at-risk Black individuals.

**Objective:**

To examine structural factors that facilitate or impede access to prostate cancer screening among Black individuals in the US.

**Design, Setting, and Participants:**

This qualitative, mixed-methods study was conducted between September 1, 2021, and December 31, 2023, in clinical and community settings across Washington, Wyoming, Alaska, Montana, Idaho, and Oregon. It included semistructured interviews with Black adults (aged ≥18 years) at risk for prostate cancer with or without a history of prostate-specific antigen (PSA) testing and a survey of primary care practitioners (PCPs) and urologists.

**Main Outcomes and Measures:**

Patient and physician experiences, knowledge, attitudes, and practices of PSA testing and prostate cancer screening were evaluated. Consensus coding and thematic analysis were used to analyze interviews; surveys were analyzed using descriptive statistics.

**Results:**

A total of 29 Black men (median [range] age, 59 [32-72] years) participated in the interviews, and 31 PCPs (including 30 phyicians and 1 physician assistant) and 32 urologists (45 of 63 aged 30-59 years [71.4%]; 40 male [63.5%]) participated in the survey. Interview participants perceived that PCPs function as gatekeepers in accessing PSA testing but may lack knowledge specific to Black men’s risk for prostate cancer and hold attitudes about PSA testing that do not support its use. Interview participants also reported a lack of trusted relationships with PCPs to support shared decision-making. While both urologists and PCPs were highly aware of US Preventive Services Task Force guidelines, PCPs were much less likely than urologists to believe in the value of PSA testing or the role of early detection to prevent prostate cancer–related mortality (2 [6.5%] vs 24 [75.0%], respectively).

**Conclusions and Relevance:**

In this qualitative study examining structural factors associated with access to prostate cancer screening among Black individuals, findings from the survey supported participants’ perceptions that PCPs do not value PSA testing for prostate cancer early detection or appreciate its role in reducing the risk of prostate cancer–related mortality. Primary care practitioner reliance on USPSTF guidelines, which currently do not provide guideline recommendations for screening high-risk groups, including Black individuals, suggests that incorporating evidence-driven guidance for PSA screening among Black individuals into these guidelines may substantially improve prostate cancer early detection among this high-risk population.

## Key Points

**Question:**

What are the patient- and physician-level factors that facilitate or impede access to prostate-specific antigen (PSA) testing for prostate cancer screening among Black individuals in the US?

**Findings:**

This qualitative study interviewing 29 Black men and surveying 63 physicians found that primary care practitioners may not value PSA testing for prostate cancer early detection or appreciate its role in reducing the risk of prostate cancer–related mortality. They may also rely on US Preventive Services Task Force guidelines for PSA testing, which currently do not provide guideline recommendations for screening high-risk groups, including Black individuals.

**Meaning:**

These findings suggest that patients and primary care practitioners need improved access to accurate and evidence-based information regarding prostate cancer risk and PSA testing among Black men.

## Teaser

This qualitative study examines patient- and physician-level structural factors that may potentially facilitate or impede access to prostate-specific antigen (PSA) testing among Black individuals at risk for prostate cancer.

## Introduction

Prostate cancer has the widest racial disparity in cancer-related death in the US, with a twofold higher risk among Black individuals compared with the average US population.^1^ This twofold higher rate of prostate cancer death has remained stable in the US over the past 5 decades, despite an overall 50% reduction following the introduction of prostate-specific antigen (PSA) testing in the late 1980s.^2,3,4^ Racial disparities in prostate cancer–related death can be attributed to a 70% to 80% higher rate of incident prostate cancer, a younger age of cancer onset, more aggressive prostate cancer at diagnosis, and worse survival among Black individuals with locoregional cancers.^5^ These epidemiologic factors are informed by structural, social, biological, environmental, and health care–related determinants of health, which all impact health behaviors and outcomes in a population.^6,7^

Despite controversies surrounding PSA-based screening, randomized clinical trial data have demonstrated that PSA testing is associated with a greater than 20% to 30% mortality reduction in prostate cancer–related death at 16 to 25 years of follow-up.^8,9,10^ In previous work, a quantitative, microsimulation model–based approach investigating whether annual PSA testing among Black individuals aged 45 to 69 years found a nearly 35% reduction in prostate cancer death.^11^ Microsimulation model analysis is needed to assess the impact of PSA testing in Black populations due to the low representation (0%-3%) of Black individuals in the PLCO and ERSPC screening trials.^12^ Unfortunately, models cannot always account for external factors that may influence prostate cancer screening patterns and behaviors among Black individuals and other marginalized, underrepresented patient populations in the US beyond the 2012 US Preventive Services Task Force (USPSTF) recommendations, which decreased screening among all races in the US.^13^

To translate guidelines and policies into actionable practice requires an understanding of how structural determinants of equity (ie, structural racism, laws and policies, economic policies), social determinants of health, and health care delivery impact prostate cancer screening among Black individuals. We hypothesized that Black individuals may experience unique barriers and facilitators to successful PSA screening practices. The aims of this mixed-methods analysis were to (1) examine the factors that facilitate or impede access to PSA testing as experienced by Black individuals and (2) to assess the knowledge, attitudes, and practices regarding PSA testing for prostate cancer screening among primary care and urology practitioners. An evaluation of these patient- and practitioner-level factors is needed to understand prostate cancer care and outcomes disparities among Black individuals.

## Methods

This qualitative study was conducted between September 1, 2021, and December 31, 2023, in the Washington, Wyoming, Alaska, Montana, Idaho, and Oregon region (WWAMI-O) in partnership with the Black and African-Descent Collaborative for Prostate Cancer Action (BACPAC), a collaboration of patient, community, advocacy, clinical, and research stakeholders with a national community advisory infrastructure that involves Black individuals as equitable partners in research. We used a mixed-methods study design in which qualitative and quantitative components were performed separately and findings were integrated into a synthesized analysis.^14,15,16^ Findings were reviewed with BACPAC advisors to aid in validating the analysis team’s interpretation of interview and survey data. This study was submitted to the University of Washington and Fred Hutchinson Cancer Center Institutional Review Board panels for ethics review, and exempt determinations with waiver of informed consent documentation were issued by both institutions as the project was designated a nonresearch activity. This study followed the Standards for Reporting Qualitative Research (SRQR) reporting guideline.^17^

### Data Collection

Interviews investigated how Black individuals experience structural and social and interpersonal barriers and facilitators related to prostate cancer screening. Participants were Black adults (aged ≥18 years) assigned male sex at birth and who resided in the Puget Sound region of Washington State. The BACPAC advisors worked closely with the study team to develop interview protocols, recruitment materials, and interview guides.^18,19^ Interview participants were recruited through email to BACPAC’s partner community organizations (eg, Puget Sound area chapters of the NAACP) and the Fred Hutchinson Cancer Center’s Office of Community Outreach and Engagement, as well as through study team member attendance at community meetings and events (eg, church-sponsored wellness events). Interviews were conducted by a study team member (D.M.) who was racially concordant with interviewees and holds expertise in cancer-related outreach and education in Black communities. All interviews were performed using Health Insurance Portability and Accountability Act–compliant, secure videoconferencing software or in person; audio recorded; and transcribed.

The survey was adapted for a US context from a large, national survey of German physicians focused on attitudes and practices around prostate cancer screening.^20^ Surveys were distributed to primary care practitioners (PCPs) and urologists in the WWAMI-O region in partnership with the Washington State Urology Society, the Washington State Medical Association, and the WWAMI-O region Practice and Research Network.

### Statistical Analysis

The interview analysis team was led by a study team member with expertise in qualitative and mixed-methods research (J.R.L.) and included 2 additional analysts (D.M. and a nonauthor) with experience in qualitative analysis. We used a prostate cancer disparities theoretical framework to guide the analysis, which proceeded iteratively, beginning with a set of a priori codes derived from the prostate cancer–specific conceptual model articulated in the theoretical framework we used (Figure).^6^ Construct-level saturation was assessed throughout the analysis process until the point at which no additional constructs pertaining to the conceptual model were identified, therefore ensuring the adequacy of the interview sample size.^21^ The analysis team used consensus coding methodology, with the lead qualitative researcher (J.R.L.) resolving nonconcordant codes.^22,23^

**Figure.**
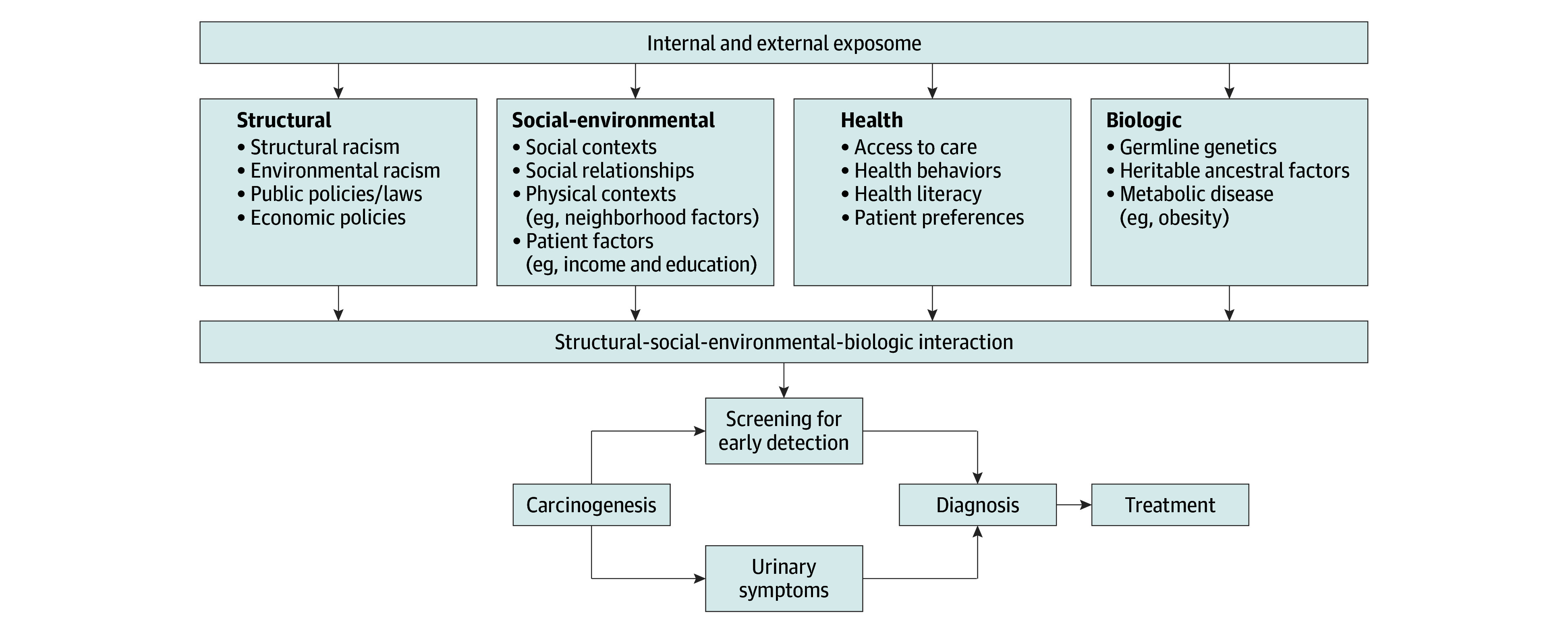
Prostate Cancer Disparities Theoretical Framework and Conceptual Model

Survey data were collected and managed using REDCap (Research Electronic Data Capture) tools hosted by the Institute of Translational Health Sciences at the University of Washington.^24,25^ Respondents were stratified by specialty (ie, primary care vs urology) for all analyses. Survey respondent data on age (<30 years, 30-39 years, 40-49 years, 50-59 years, 60-69 years, and ≥70 years), sex assigned at birth, self-selected race (American Indian or Alaska Native, Asian, Black, White, multiracial) and ethnicity (Hispanic or Latinx or non-Hispanic or Latinx), degree, work experience and setting, and practice type were collected and used to describe the sample. We performed descriptive statistical analyses using REDCap but did not perform any quantitative comparative analyses due to the small sample sizes of our 2 cohorts.

## Results

Twenty-nine non-Hispanic or Latinx Black men participated in interviews between September 14, 2021, and March 29, 2022. The median age of participants was 59 years (range, 32-72 years), 21 (72.4%) had undergone at least 1 prior PSA test for prostate cancer screening, and 14 (66.7%) of those screened had been diagnosed with prostate cancer (eTable 2 in Supplement 1). Survey respondents included 31 PCPs (30 physicians and 1 physician assistant) and 32 urologists (1 of 63 aged ≤30 years [1.6%], 45 aged 30-59 years [71.4%] and 17 aged ≥60 years [26.9%]; 23 female [36.5%] and 40 male [63.5%]; 1 identifying as American Indian or Alaska Native [1.6%], 10 identifying as Asian [15.9%]; 2 as Black [3.2%]; and 52 as White [82.5%] race; 2 identifying as multiracial [3.2%]; 4 identifying as Hispanic or Latinx [6.4%] and 59 as not Hispanic or Latinx [93.7%]), of whom 31 (49.2%) were PCPs (including 1 physician assistant) and 32 (50.8%) were urologists (eTable 2 in Supplement 1).

Interviews queried participants about their knowledge and experiences regarding prostate cancer and PSA testing. Surveys queried physicians about their knowledge of and reliance on national prostate cancer screening practice guidelines, use of PSA testing in clinical practice, and knowledge and attitudes regarding the utility of PSA testing for prostate cancer early detection (eTable 1 in Supplement 1). The integrated analysis revealed 3 key findings that highlight the critical interplay among structural, social, and health care delivery factors that influence Black individuals’ access to PSA testing. These integrated findings were the (1) lack of prostate cancer resources for Black patients and their physicians, (2) perception among PCPs about the benefit of PSA testing, and (3) understanding of patient lived experiences and values by PCPs.

### Integrated Finding 1: Access to PSA Testing and Availability of Information on Prostate Cancer Risk

Both interviews and surveys showed a lack of readily available information about prostate cancer screening and PSA testing that directly addresses Black individuals as a population at risk. Interview participants noted that dominant narratives regarding Black individuals’ health often revolve around risk for cardiovascular disease and diabetes, while prostate cancer remains relatively obscure (Table 1). Participants reported that information on other medical conditions was more readily available than information about prostate cancer, both in discussion with physicians and in materials available in their physician’s office. This lack of information persisted both in community settings and within the health care delivery system, wherein patients may not be made aware of the availability of prostate cancer screening until they present with urinary symptoms.

**Table 1.** Semistructured Interview Results by Integrated Finding

Exemplary quote	Participant age, y	Underwent PSA screening	History of prostate cancer
**Integrated finding 1: access to PSA testing is associated with availability of information about Black individuals’ prostate cancer risk**			
“We talk more about cholesterol, heart attacks, strokes, high blood pressure…for African Americans especially….But then again, when you say prostate cancer for men, it’s not as out in the forefront as these other causes of death in minorities.”	60s	Yes	Yes
“That is information that should definitely be put out there to the young African Americans so that we know, ‘Hey, this is something you need to stay focused on because this can happen because it does run strong in African Americans.’ That’s information that’s never been put out there. And that’s why I was shocked when they told me I had it. I’m like, ‘What? Prostate cancer, what is that?’ Because no one ever said, ‘This is something that runs strong in African Americans.’ It’s not the kind of information that they put out there on a ready basis.”	60s	Yes	Yes
“I’ve never really known anybody that has been afflicted with [prostate cancer], so I’m not very knowledgeable in terms of its effect on people; who gets it, how they get it, what age you’re most likely to get it. I have very little information regarding that….What ages you should be tested for it; what are the likely symptoms to look for; is it more ethnic related; or is it just a male thing across the board, regardless of ethnicity, like high blood pressure and diabetes being more to certain ethnic races are more susceptible to it? Is it that kind of scenario?”	NA	No	No
**Integrated finding 2: access to PSA testing is associated with physician perception of its value and utility**			
“I wanted to have a PSA test in January…when I asked [the physician] for the test, he said it wasn’t necessary. He didn’t want to put me through any unnecessary procedures….And then about November, I had a little incontinence for about 2 weeks….And once I took the [PSA] test, that’s when they found out my [PSA] numbers were extremely high...that’s when we discovered I was at stage IV.”	70s	Yes	Yes
“The particular doctor that I had, I feel like he let me down. I feel like he’s done me a grave injustice because…not once did he suggest or send me to a urologist to have my prostate checked. I didn’t get my prostate checked until I switched doctors because I felt like the doctor that I had wasn’t doing anything for me….And when I met the doctor that I have now, I told him flat out. I said, ‘Look, man, something’s wrong with me. I don’t know what it is, but I’m positive something is wrong with me because I’m always tired.’ And I said, ‘Lately, I’ve been urinating like crazy.’ I said, ‘I haven’t had a decent checkup since I can remember.’ So he said, ‘The first thing we’re going to do is we’re going to take a lot of blood, run a bunch of tests, and in the meantime you’re going to go see a urologist.’ And that’s how I found out what I got. That’s how I found out I got prostate cancer.”	50s	Yes	Yes
**Integrated finding 3: access to PSA testing is associated with a patient-physician relationship based on mutual trust**			
“I made it a point to get a Black primary care doctor. Serious point, I had relocated here maybe a year before I saw her. And the major thing was I wanted a Black primary care doctor. My personal feeling is if my primary care wasn’t Black, well then this is just a crapshoot, may as well go in an emergency department if something happens....The medical field, my experience with it is, it’s as rife with racism as everywhere else.”	60s	Yes	No
“I know there’s not many Black medical providers in the field. But if we can get the word out more that this is important for us as Black men to get screened and have the test, man, I think more guys will opt to have that done.”	50s	Yes	Yes
“Some disparities within health care that make it a little bit more difficult for people of color to get the types of resources or information, or have the trust that they need, having doctors and nurses who look like you to discuss those concerns. Or to point out and make the connection and make you feel comfortable that they have your best interest in mind…I’m really big on the whole relationship piece of everything, saying no real learning occurs without real relationships.”	50s	No	No
“In fact, I just read an article…about Black men’s health. And it states that when Black men have Black health providers, their health conditions improve. And that makes so much sense to me….So yeah, that’s becoming a very important issue to me….Preference sounds like a softer word, it’s becoming a requirement. I mean, I’ve already required now that my physicians be physicians of color. Whenever I can make that happen.”	60s	Yes	No

Abbreviations: NA, not available; PSA, prostate-specific antigen.

To understand sources informing physicians’ use of PSA testing, the survey queried physicians about knowledge of and reliance on USPSTF, American Urologic Association (AUA), and American Cancer Society (ACS) prostate cancer screening guidelines. Current AUA and ACS guidelines include considerations for testing individuals of populations at higher risk of being diagnosed and/or dying of prostate cancer, which includes Black individuals (eTable 3 in Supplement 1). Current USPSTF guidelines do not include recommendations specific to Black individuals (eTable 3 in Supplement 1). Primary care practitioners and urologists showed differences in their familiarity with clinical guidelines and the primary screening trials that support their recommendations (Table 2).

**Table 2.** Integrated Finding 1 Survey Results

Access to PSA testing is mediated by availability of information about Black individuals’ prostate cancer risk	No. (%)	
	PCPs (n = 31)	Urologists (n = 32)
**Are you aware of the 2018 US Preventive Services Task Force guidelines regarding routine PSA testing?**		
Have not heard of them	1 (3.2)	2 (6.3)
Have heard of them but not familiar with recommendations	5 (16.1)	6 (18.8)
Have heard of them and familiar with recommendations	25 (80.6)	24 (75.0)
**Do you agree with the 2018 US Preventive Services Task Force recommendation for prostate cancer screening?** ^a^		
Not at all agree	0	7 (23.3)
Partially agree	12 (40.0)	20 (66.7)
Completely agree	14 (46.7)	0
Unsure	4 (13.3)	3 (10.0)
**Are you aware of the 2021 American Cancer Society guidelines regarding routine PSA testing?**		
Have not heard of them	15 (48.4)	12 (37.5)
Have heard of them but not familiar with recommendations	12 (38.7)	8 (25.0)
Have heard of them and familiar with recommendations	4 (12.9)	12 (37.5)
**Do you agree with the 2021 American Cancer Society recommendation for prostate cancer screening?** ^b^		
Not at all agree	1 (6.3)	0
Partially agree	5 (31.3)	10 (50.0)
Completely agree	0 (0)	4 (20.0)
Unsure	10 (62.5)	6 (30.0)
**Are you aware of the 2018 American Urological Association guidelines regarding routine PSA testing?**		
Have not heard of them	17 (54.8)	2 (6.3)
Have heard of them but not familiar with recommendations	10 (31.3)	2 (6.3)
Have heard of them and familiar with recommendations	4 (12.9)	28 (87.5)
**Do you agree with the 2018 American Urological Association recommendation for prostate cancer screening?** ^c^		
Not at all agree	3 (21.4)	0
Partially agree	3 (21.4)	17 (56.7)
Completely agree	1 (7.1)	13 (43.3)
Unsure	7 (50.0)	0
**To what extent do the recommendations from the US Preventive Services Task Force influence your use of PSA testing?**		
Not at all	1 (3.2)	10 (31.3)
Weakly	0	12 (37.5)
Moderately	7 (22.6)	9 (28.1)
Strongly	12 (38.7)	1 (3.1)
Very strongly	11 (35.5)	0
**To what extent do the recommendations from other guidelines (ie, American Academy of Family Practice, American Urological Association, American Cancer Society) influence your use of PSA testing?**		
Not at all	4 (12.9)	2 (6.3)
Weakly	11 (35.5)	1 (3.1)
Moderately	15 (48.4)	12 (37.5)
Strongly	1 (3.2)	14 (43.8)
Very strongly	0	3 (9.4)

Abbreviations: PCP, primary care practitioner; PSA, prostate-specific antigen.

^a^ Responses missing from 1 PCP and 1 urologist.

^b^ Responses missing from 15 PCPs and 12 urologists.

^c^ Responses missing from 17 PCPs and 12 urologists.

Primary care practitioners were considerably more likely than urologists to report relying on USPSTF guidelines (30 [96.8%] vs 10 [31.2%], respectively) and much less likely than urologists to report relying on AUA or ACS guidelines (16 [51.6%] vs 29 [90.6%], respectively) to support their screening practices. Few PCPs (4 [13.0%]) reported being familiar with the content of either AUA or ACS guidelines (Table 2).

### Integrated Finding 2: Access to PSA Testing by Physician Perception of Its Value and Utility

Interview participants reported that they discussed and accessed PSA testing through their PCPs. Yet, interview participants reported that their PCPs either did not initiate conversations about PSA testing or were reluctant to order a PSA test when requested by patients (Table 1). In these instances, participants described physicians as dismissive of their concerns and prioritizing avoidance of possible negative consequences of PSA testing (eg, biopsy with a benign finding, treatment adverse effects) over the benefits of screening. Primary care practitioners were described by interview participants as not having adequate knowledge of Black individual’s prostate cancer risks when they denied them access to PSA testing. Interview participants also reported that PCPs who did not order PSA testing seemed to hold negative views of the value and utility of prostate cancer screening more broadly. Interview participants who described their PCPs as knowledgeable about Black individual’s prostate cancer risks reported that their physicians either initiated conversations about PSA testing or were supportive of screening when it was requested by the patient.

Survey findings correlated with the experiences reported by interview participants. While PCPs and urologists reported similar rates of belief that early detection is important for reducing all-cancer mortality (25 [80.6%] vs 27 [84.4%], respectively), PCPs were less likely to report that they believed early detection is important in reducing prostate cancer–specific mortality (13 [41.9%] vs 26 [81.3%], respectively) (Table 3). When asked about prostate cancer early detection, PCPs were less likely than urologists to believe that PSA testing is an overall valuable test (15 [48.4%] vs 31 [96.9%], respectively). When asked specifically about the role of PSA testing in reducing prostate cancer–specific mortality, PCPs were less likely than urologists to report a belief that PSA can substantially reduce prostate cancer death in screened patients (2 [6.5%] vs 24 [75.0%], respectively). The survey also asked physicians about the frequency with which they discussed PSA testing with their at-risk patients. The gap between PCPs and urologists was most evident in responses regarding PSA testing among populations with a higher incidence of prostate cancer in the US. Primary care practitioners were less likely than urologists to report often or always discussing PSA testing with Black patients (19 [61.3%] vs 28 [87.5%], respectively) and patients with a family history of prostate cancer (14 [45.2%] vs 29 [90.6%]).

**Table 3.** Integrated Finding 2 Survey Results

Access to PSA testing is mediated by physician perception of its value and utility	No. (%)	
	PCPs (n = 31)	Urologists (n = 32)
**In general, how important do you think early detection is for reducing death from any cancer?**		
Very unimportant	0	2 (6.3)
Unimportant	1 (3.2)	0
Undecided	5 (16.1)	3 (9.4)
Important	13 (41.9)	17 (53.1)
Very important	12 (38.7)	10 (31.3)
**How important do you think early detection is for reducing death from prostate cancer?**		
Very unimportant	1 (3.2)	1 (3.1)
Unimportant	4 (12.9)	1 (3.1)
Undecided	13 (41.9)	4 (12.5)
Important	9 (29.0)	17 (53.1)
Very important	4 (12.9)	9 (28.1)
**What is your understanding of the value of PSA testing in general?**		
Not at all valuable	0	1 (3.1)
Not valuable	5 (16.1)	0
Undecided	11 (35.5)	0
Valuable	15 (48.4)	28 (87.5)
Very valuable	0	3 (9.4)
**Do you think PSA testing causes a significant reduction in the chance that a man will die of prostate cancer?**		
No, clearly not	20 (64.5)	1 (3.1)
Undecided	9 (29.0)	7 (21.9)
Yes, clearly proven	2 (6.5)	24 (75.0)
**How often do you discuss PSA testing with a patient who has a family history of prostate cancer?**		
Never	0	1 (3.1)
Rarely	0	0
Sometimes	6 (30.0)	0
Often	8 (40.0)	4 (12.5)
Always	6 (30.0)	25 (78.1)
Not applicable	0	2 (6.3)
**How often do you discuss PSA testing with a patient who identifies as Black or African American?**		
Never	2 (6.5)	1 (3.2)
Rarely	1 (3.2)	0
Sometimes	7 (22.6)	1 (3.2)
Often	11 (35.5)	9 (28.1)
Always	8 (25.8)	19 (59.4)
Not applicable	2 (6.5)	2 (6.3)

Abbreviations: PCP, primary care practitioner; PSA, prostate-specific antigen.

### Integrated Finding 3: Access to PSA Testing Based on Mutual Trust

A trusted relationship with physicians is a critical factor that influenced access to PSA testing among interview participants. An important factor in establishing rapport and trust with a physician was described by participants as the ability to have their lived experiences as Black individuals sufficiently understood by their physicians without the patient bearing the responsibility for establishing that level of understanding in the patient-physician relationship (Table 1). Participants reported a strong preference for physicians who understand the historical and current-day contexts of racism, marginalization, and disenfranchisement that have a direct impact on Black individual’s experiences of health and health care. In some cases, this preference led participants to exclusively seek out Black physicians because they viewed these individuals as having critical shared experience and understanding. However, Black physicians were often not readily available, which led some participants to access care from physicians who hold other historically marginalized identities. Other participants reported comfort with physicians of any racial or ethnic identity as long as they felt that the physician held an adequate understanding of the patient’s lived experience and cultural values and norms. The difficulty in finding physicians of races other than White was reflected in our survey sample (28 PCPs [90.3%] and 24 urologists [75.0%] self-identifying as White compared with 1 PCP [3.2%] and 1 urologist [3.1%] self-identifying as Black) (Table 4).

**Table 4.** Integrated Finding 3 Survey Results

Access to PSA testing is mediated by a patient-physician relationship based on mutual trust	No. (%)	
	PCPs (n = 31)	Urologists (n = 32)
Physician race^a^		
American Indian or Alaska Native	0	1 (3.1)
Asian	4 (12.9)	6 (18.8)
Black or African American	1 (3.2)	1 (3.1)
White	28 (90.3)	24 (75.0)
Multiracial	0	2 (6.3)
Physician ethnicity		
Hispanic or Latinx	2 (6.5)	2 (6.3)
Not Hispanic or Latinx	29 (93.5)	30 (93.7)

Abbreviations: PCP, primary care practitioner; PSA, prostate-specific antigen.

^a^ Respondents were able to select more than 1 option.

## Discussion

This mixed-methods qualitative study evaluated the barriers and facilitators of PSA testing among Black men, illuminating key aspects of patient- and physician-level factors that contribute to whether Black individuals receive PSA testing for the early detection of prostate cancer. Black individuals have experienced a consistent and dramatic disparity in prostate cancer death in the US over the past 50 years, despite advances in the diagnosis and treatment of prostate cancer.^2,3,4^ Level 1 evidence demonstrates a benefit to PSA screening,^8,10^ but previous studies have shown that rates of PSA screening have declined among Black individuals following the 2012 USPSTF PSA screening recommendation.^13^

Our first integrated finding suggests that both patients and physicians come to informed decisions around PSA-based screening with substantial knowledge deficits regarding benefits and harms of prostate cancer early detection. Patients and clinician may lack awareness of the disparate incidence and mortality outcomes of prostate cancer among Black individuals. There is limited information for Black patients about prostate cancer, and the information that is available is often inaccessible and of low quality.^26^ While current guidelines vary in their recommendations for prostate cancer screening, all recommend shared decision-making between patient and clinician as foundational in determining which patients should ultimately receive PSA testing. Survey responses clearly highlighted that PCPs relied on the USPSTF guidelines to inform their practice around PSA testing. In contrast to AUA and ACS guidelines, which provide evidence-based recommendations for earlier screening in high-risk populations, the USPSTF guidelines do not provide specific guidance for populations at higher risk for prostate cancer incidence and/or death.^27,28^

The PCPs we surveyed reported low levels of confidence in the value of PSA testing for reducing prostate cancer death. Differences in guideline recommendations over time have impacted the views and use of PSA testing among PCPs.^29^ In our sample, PCPs consistently reported low confidence in PSA testing in reducing prostate cancer death and lack of familiarity with the 2 prospective screening trials that assessed the efficacy of PSA testing. Primary care practitioners also reported a substantially lower likelihood than urologists of discussing PSA testing with Black patients and with patients who have a family history of prostate cancer. This finding suggests that PCPs may lack general knowledge around higher risk populations for incident and fatal prostate cancer. It is important to note that the long-term benefit of PSA testing is a 20% to 30% reduction in prostate cancer death.^8,9,10^ However, this benefit must be weighed against the harms of screening, which include false-positive test results, diagnostic uncertainty, overdetection, overtreatment, and adverse effects of both diagnostic and therapeutic procedures. A recent guideline recommendation that was supported by a systematic review was published by the Prostate Cancer Foundation and provides evidenced-based recommendations for screening among Black patients.^7^ These contemporary data and recommendations should be considered by PCPs engaging in informed decision-making with Black patients in their practices.

Decision-making for prostate cancer screening requires discussion with physicians about potentially sensitive topics. Such discussions could pose barriers to holding shared decision-making discussions if patients do not feel sufficient rapport and trust in their physicians. Interview participants reported successful informed decision-making about PSA testing with physicians, largely in the setting of care from racially or culturally concordant PCPs. Previous studies have shown that racially concordant health care is associated with improved clinical outcomes, communication, patient satisfaction, communication, and therapeutic relationships for underrepresented patients in the US.^30,31,32^ The racial distribution of survey respondents mirrors that of the medical workforce in the US, in which Black physicians represent only 5% of the physician workforce.^33^ There are a number of ongoing efforts to increase and grow the pipeline of Black physicians, starting as early as in high school,^34,35,36^ which should be supplemented with medical education that emphasizes the importance of understanding and acknowledging the lived experiences and cultural norms of patients, especially those from marginalized communities.

### Limitations

This study has some limitations. First, it was limited by our geographic sample, which was drawn from the northwestern region of the US. However, many of our findings are consistent with other studies that evaluated the association between race and health care use.^30,31,32^ Second, a higher proportion of interview participants reported a history of prostate cancer than the overall proportion of Black individuals who will develop prostate cancer in their lifetimes (48% vs approximately 17%).^37^ Third, the study was limited by our survey sampling approach, which did not allow us to calculate a survey response rate. Finally, we recognize that Black individuals represent a diverse and heterogeneous population of the US in terms of social and economic status, ethnicities, and cultural norms and that our sample of interviewees may not represent all of these nuances within the population. However, we must also emphasize that Black individuals’ lived experience shares many commonalities that transcend social strata and cultural values and norms.

## Conclusions

This mixed-methods qualitative study of structural factors associated with access to prostate cancer screening among Black individuals found that these individuals had unique needs around the early detection of prostate cancer, which we must meet to address the crisis of disparate prostate cancer death among Black men. Findings from the survey suggest that PCPs do not value PSA testing for prostate cancer early detection or appreciate its role in reducing the risk of prostate cancer–related death. The study also found that PCPs were more likely than urologists to be influenced by guidelines that do not provide specific recommendations for screening high-risk populations, including Black individuals. Incorporating evidence-driven guidance for PSA screening among Black individuals into the guidelines that PCPs rely on could therefore substantially improve prostate cancer early detection among this highly at-risk population.

## Notes

Supplement 1. **eTable 1.** Full Survey Results **eTable 2.** Demographics of Survey Respondents and Interview Participants **eTable 3.** Prostate Cancer Screening Recommendations

Supplement 2.
Data Sharing Statement
