# Supplementary material for: Patient and Physician Perceptions of Prostate-Specific Antigen Testing Among Black Individuals
Source: JAMA Netw Open. 2025 Sep 8;8(9):e2530946. doi: 10.1001/jamanetworkopen.2025.30946 (PMC12418120; doi:10.1001/jamanetworkopen.2025.30946)
Supplement: Supplement 1. — eTable 1. Full Survey Results eTable 2. Demographics of Survey Respondents and Interview Participants eTable 3. Prostate Cancer Screening Recommendations [file jamanetwopen-e2530946-s001.pdf]

Supplemental Online Content

Lee JR, Morehead D, Young B, et al. Patient- and physician-level factors in prostate-specific antigen testing among Black American men. *JAMA Netw Open*. 2025;8(9):e2530946. doi:10.1001/jamanetworkopen.2025.30946

eTable 1. Full Survey Results

eTable 2. Demographics of Survey Respondents and Interview Participants

eTable 3. Prostate Cancer Screening Recommendations

This supplemental material has been provided by the authors to give readers additional information about their work.

© 2025 Lee JR et al. *JAMA Network Open*.

eTable 1. Full Survey Results

	Primary Care Providers (n=31)		Urologists (n=32)	
	n	%	n	%
Are you aware of the 2018 US Preventive Services Task Force guidelines regarding routine PSA testing?				
Have not heard of them	1	3%	2	6%
Have heard of them, but not familiar with recommendations	5	16%	6	19%
Have heard of them and familiar with recommendations	25	81%	24	75%
Do you agree with the 2018 US Preventive Services Task Force recommendation for prostate cancer screening?[1]				
Completely agree	14	47%	0	0%
Partially agree	12	40%	20	67%
Not at all agree	0	0%	7	23%
Unsure	4	13%	3	10%
Are you aware of the 2021 American Cancer Society guidelines regarding routine PSA testing?				
Have not heard of them	15	48%	12	38%
Have heard of them, but not familiar with recommendations	12	39%	8	25%
Have heard of them and familiar with recommendations	4	13%	12	38%
Do you agree with the 2021 American Cancer Society recommendation for prostate cancer screening?[1]				
Completely agree	0	0%	4	20%
Partially agree	5	31%	10	50%
Not at all agree	1	6%	0	0%
Unsure	10	63%	6	30%
Are you aware of the 2018 American Urological Association guidelines regarding routine PSA testing?				
Have not heard of them	17	55%	2	6%
Have heard of them, but not familiar with recommendations	10	32%	2	6%
Have heard of them and familiar with recommendations	4	13%	28	88%
Do you agree with the 2018 American Urological Association recommendation for prostate cancer screening?[1]				
Completely agree	1	7%	13	43%

© 2025 Lee JR et al. *JAMA Network Open*.

Partially agree	3	21%	17	57%
Not at all agree	3	21%	0	0%
Unsure	7	50%	0	0%
Are you aware of the results from the Prostate, Lung, Colon, and Ovarian (PLCO) PSA screening trial?				
Have not heard of them	23	74%	4	13%
Have heard of them, but not familiar with findings	4	13%	9	28%
Have heard of them and familiar with findings	4	13%	19	59%
Do you agree with the validity of the conclusion of the PLCO PSA screening trial? [1]				
Completely agree	0	0%	3	16%
Partially agree	2	50%	4	21%
Not at all agree	0	0%	11	58%
Unsure	2	50%	1	5%
Are you aware of the results from the European Randomized Study of Prostate Cancer (ERSPC) PSA screening trial?				
Have not heard of them	24	77%	6	19%
Have heard of them, but not familiar with findings	3	10%	9	28%
Have heard of them and familiar with findings	4	13%	17	53%
Do you agree with the validity of the conclusion of the ERSPC PSA screening trial? [1]				
Completely agree	1	25%	6	35%
Partially agree	2	50%	10	59%
Not at all agree	0	0%	1	6%
Unsure	1	25%	0	0%
To what extent do the recommendations from the US Preventive Services Task Force influence your use of PSA testing?				
Not at all	1	3%	10	31%
Weakly	0	0%	12	38%
Moderately	7	23%	9	28%
Strongly	12	39%	1	3%
Very strongly	11	35%	0	0%

© 2025 Lee JR et al. *JAMA Network Open*.

To what extent do the recommendations from other guidelines (i.e., American Academy of Family Practice, American Urological Association, American Cancer Society) influence your use of PSA testing?				
Not at all	4	13%	2	6%
Weakly	11	35%	1	3%
Moderately	15	48%	12	38%
Strongly	1	3%	14	44%
Very strongly	0	0%	3	9%
To what extent do results of the Prostate, Lung, Colon, and Ovarian (PLCO) trial screening trial influence your use of PSA testing?				
Not at all	19	61%	14	44%
Weakly	5	16%	10	31%
Moderately	6	19%	7	22%
Strongly	1	3%	1	3%
Very strongly	0	0%	0	0%
To what extent do results of the European Randomized Study of Prostate Cancer (ERSPC) trial screening trial influence your use of PSA testing?				
Not at all	19	61%	9	28%
Weakly	5	16%	3	9%
Moderately	7	23%	12	38%
Strongly	0	0%	7	22%
Very strongly	0	0%	1	3%
In general, how important do you think early detection is for reducing death from any cancer?				
Very unimportant	0	0%	2	6%
Unimportant	1	3%	0	0%
Undecided	5	16%	3	9%
Important	13	42%	17	53%
Very important	12	39%	10	31%
How important do you think early detection is for reducing death from prostate cancer?				
Very unimportant	1	3%	1	3%
Unimportant	4	13%	1	3%

© 2025 Lee JR et al. *JAMA Network Open*.

Undecided	13	42%	4	13%
Important	9	29%	17	53%
Very important	4	13%	9	28%
What is your understanding of the value of PSA testing in general?				
Not at all valuable	0	0%	1	3%
Not valuable	5	16%	0	0%
Undecided	11	35%	0	0%
Valuable	15	48%	28	88%
Very valuable	0	0%	3	9%
Have you (personally) ever had a PSA test?				
No	16	52%	16	50%
Yes	7	23%	14	44%
Not applicable	8	26%	2	6%
Do you plan to undergo PSA testing in the future?				
Definitely not	2	6%	5	16%
Likely not	4	13%	2	6%
Undecided	3	10%	2	6%
Possibly	6	19%	0	0%
Definitely	3	10%	18	56%
Not applicable	13	42%	5	16%
Would you recommend a PSA test for the early detection of prostate cancer to a loved one (e.g. partner, father or brother)?				
Definitely not	1	3%	1	3%
Likely not	6	19%	0	0%
Undecided	8	26%	1	3%
Possibly	12	39%	2	6%
Definitely	4	13%	28	88%
Do you think PSA testing causes a significant reduction in the chance that a man will die from prostate cancer?				
No, clearly not	20	65%	1	3%
Undecided	9	29%	7	22%
Yes, clearly proven	2	6%	24	75%
Is there a standard procedure regarding PSA testing (in your practice)?				

© 2025 Lee JR et al. *JAMA Network Open*.

Yes	12	39%	9	28%
No	17	55%	16	50%
Unknown	0	0%	5	16%
Not applicable	2	6%	2	6%
When was this standard last updated? [1]				
≤ 3 years	7	58%	5	56%
4-9 years	3	25%	2	22%
≥ 10 years	0	0%	0	0%
Don't know	2	17%	2	22%
Not applicable	0	0%	0	0%
Who is responsible for ordering and interpreting PSA tests in your practice?				
Physician	31	100%	30	94%
Medical Assistant	0	0%	0	0%
No specific person	0	0%	0	0%
No person/consultation not performed	0	0%	0	0%
Other	0	0%	1	3%
Not applicable	0	0%	1	3%
How do you ask patients if they would like a PSA test performed during their visit?				
I don't not ask	0	0%	1	3%
Face to face discussion	31	100%	31	97%
Standardized written form	0	0%	0	0%
Other	0	0%	0	0%
How are discussions or consultations for PSA testing performed in your practice?				
Face to face discussion	29	94%	32	100%
Give patient written material with discussion	1	3%	0	0%
Give patient written material without discussion	0	0%	0	0%
Written material available in waiting room	0	0%	0	0%
Other	1	3%	0	0%
Not discussed	0	0%	0	0%
Has your practice changed around routine PSA testing in the last ten years?				
Yes	14	45%	19	59%
No	11	35%	9	28%

© 2025 Lee JR et al. *JAMA Network Open*.

Unknown	4	13%	1	3%
Not applicable	2	6%	3	9%
In which direction has your practice changed PSA testing? I perform PSA tests ... than I did ten years ago. [1]				
Much less frequently	2	14%	2	11%
Less frequently	8	57%	12	63%
More frequently	3	21%	3	16%
Much more frequently	0	0%	0	0%
Not applicable	1	7%	2	11%
What is the minimum life expectancy (years) an asymptomatic patient needs to have for you to recommend or consider PSA testing?				
I recommend it regardless of life expectancy	0	0%	1	3%
5-9 years	3	10%	6	19%
10-14 years	21	68%	23	72%
≥15 Years	4	13%	2	6%
I never recommend it	3	10%	0	0%
Not applicable	0	0%	0	0%
What proportion of men aged 45-55 years in your practice receive an annual or biennial PSA test?				
Almost none	15	48%	8	25%
About one quarter	9	29%	5	16%
About half	3	10%	5	16%
About three quarters	1	3%	3	9%
Almost all	1	3%	6	19%
Not applicable	2	6%	5	16%
What proportion of men aged 55-70 years in your practice receive an annual or biennial PSA test?				
Almost none	5	16%	1	3%
About one quarter	14	45%	3	9%
About half	7	23%	4	13%
About three quarters	1	3%	6	19%
Almost all	2	6%	15	47%
Not applicable	2	6%	3	9%
What proportion of men aged 70 years and older in your practice receive an annual or biennial PSA test?				

© 2025 Lee JR et al. *JAMA Network Open*.

Almost none	23	74%	3	9%
About one quarter	2	6%	11	34%
About half	4	13%	8	25%
About three quarters	1	3%	7	22%
Almost all	0	0%	0	0%
Not applicable	1	3%	3	9%
Where do PSA blood draws occur in your practice?				0%
Onsite at our clinic	29	94%	17	53%
At an outside laboratory	2	6%	10	31%
Other	0	0%	3	9%
Not applicable	0	0%	2	6%
Case scenario 1: Imagine you see an asymptomatic man under the age of 70 without risk factors. Would you recommend that he consider a PSA test within a certain age range?				
Yes	16	52%	31	97%
No	15	48%	1	3%
Case scenario 2: Imagine you see a 45-year-old patient with a life expectancy of at least 10 years who does not ask for a PSA test. Would you actively discuss or recommend PSA testing with him?				
Yes	9	29%	10	31%
No	22	71%	22	69%
Case scenario 3: Imagine you see a 45-year-old patient in your practice with a life expectancy of at least 10 years has a PSA level of 1-2 ng/mL. At what interval would you recommend for a PSA test moving forward?				
Every year or more often	8	26%	9	28%
Every 2 years	10	32%	17	53%
Every 3 years	2	6%	2	6%
Every 4 years	2	6%	1	3%
Longer than every 4 years	3	10%	2	6%
Not at all	6	19%	1	3%
HOW OFTEN DO YOU DISCUSS PSA TESTING…				0%
As part of general cancer screening for a man in your practice?				

© 2025 Lee JR et al. *JAMA Network Open*.

Never	0	0%	1	3%
Rarely	5	16%	2	6%
Sometimes	6	19%	3	9%
Often	11	35%	15	47%
Always	9	29%	8	25%
Not applicable	0	0%	3	9%
With a patient who has a family history of prostate cancer?				
Never	0	0%	1	3%
Rarely	0	0%	0	0%
Sometimes	6	19%	0	0%
Often	8	26%	4	13%
Always	6	19%	25	78%
Not applicable	0	0%	2	6%
With a patient who has a family history of breast, ovarian, or colon cancer?				
Never	3	10%	1	3%
Rarely	2	6%	0	0%
Sometimes	10	32%	2	6%
Often	10	32%	9	28%
Always	6	19%	18	56%
Not applicable	0	0%	2	6%
With a patient who identifies as Black or African American?				
Never	2	6%	1	3%
Rarely	1	3%	0	0%
Sometimes	7	23%	1	3%
Often	11	35%	9	28%
Always	8	26%	19	59%
Not applicable	2	6%	2	6%
With a patient who has discomfort of the lower urinary tract (i.e., bladder, urethra)?				
Never	0	0%	1	3%
Rarely	3	10%	3	9%
Sometimes	6	19%	5	16%
Often	10	32%	14	44%
Always	12	39%	7	22%
Not applicable	0	0%	2	6%

© 2025 Lee JR et al. *JAMA Network Open*.

HOW OFTEN DO YOU DISCUSS THE FOLLOWING ASPECTS OF PSA TESTING WITH YOUR PATIENTS WHO ARE CONSIDERING PROSTATE CANCER SCREENING?				
Benefits of PCa early detection (i.e., reducing the risk of death from prostate cancer)				
Never	1	3%	1	3%
Rarely	3	10%	1	3%
Sometimes	5	16%	2	6%
Often	10	32%	10	31%
Always	12	39%	16	50%
Not applicable	0	0%	2	6%
Possible risks of PCa early detection (i.e., overdiagnosis of indolent cancer, overtreatment of indolent cancers)				
Never	0	0%	1	3%
Rarely	0	0%	1	3%
Sometimes	2	6%	1	3%
Often	10	32%	5	16%
Always	19	61%	22	69%
Not applicable	0	0%	2	6%
The possibility of a 'false positive' PSA test (i.e., elevated PSA with negative prostate biopsy)				
Never	0	0%	2	6%
Rarely	0	0%	1	3%
Sometimes	2	6%	0	0%
Often	11	35%	5	16%
Always	18	58%	22	69%
Not applicable	0	0%	2	6%
Potential anxiety associated with screening and biopsy				
Never	0	0%	3	9%
Rarely	2	6%	2	6%
Sometimes	2	6%	5	16%
Often	12	39%	9	28%
Always	15	48%	12	38%
Not applicable	0	0%	2	6%
The need for more testing or procedures for elevated PSA				
Never	0	0%	2	6%

© 2025 Lee JR et al. *JAMA Network Open*.

Rarely	0	0%	1	3%
Sometimes	2	6%	1	3%
Often	10	32%	7	22%
Always	19	61%	19	59%
Not applicable	0	0%	2	6%
Options for and adverse effects of treatment for localized prostate cancer (i.e., observation, radiation, surgery and impact on sexual, urinary, and bowel function)				
Never	4	13%	3	9%
Rarely	4	13%	0	0%
Sometimes	4	13%	5	16%
Often	8	26%	11	34%
Always	11	35%	11	34%
Not applicable	0	0%	2	6%
Prostate imaging in aiding diagnosis (i.e., prostate MRI)				0%
Never	12	39%	3	9%
Rarely	7	23%	0	0%
Sometimes	5	16%	6	19%
Often	4	13%	12	38%
Always	3	10%	9	28%
Not applicable	0	0%	2	6%
HOW OFTEN DO YOU PERFORM A DIGITAL RECTAL EXAMINATION IN THE FOLLOWING SITUATIONS?				
During an early cancer detection examination				
Never	17	55%	1	3%
Rarely	8	26%	2	6%
Sometimes	3	10%	3	9%
Often	1	3%	5	16%
Always	2	6%	18	56%
Not applicable	0	0%	3	9%
If there is blood in the patient's stool				
Never	4	13%	3	9%
Rarely	2	6%	2	6%
Sometimes	7	23%	3	9%
Often	6	19%	3	9%
Always	12	39%	10	31%

© 2025 Lee JR et al. *JAMA Network Open*.

Not applicable	0	0%	11	34%
If the patient has a voiding disorder				
Never	5	16%	1	3%
Rarely	6	19%	0	0%
Sometimes	10	32%	3	9%
Often	6	19%	14	44%
Always	4	13%	13	41%
Not applicable	0	0%	1	3%
If the patient is asymptomatic				
Never	22	71%	3	9%
Rarely	6	19%	5	16%
Sometimes	2	6%	10	31%
Often	1	3%	8	25%
Always	0	0%	4	13%
Not applicable	0	0%	2	6%
If the patient has an elevated PSA				
Never	5	16%	1	3%
Rarely	4	13%	0	0%
Sometimes	8	26%	2	6%
Often	7	23%	6	19%
Always	7	23%	21	66%
Not applicable	0	0%	2	6%
Which of the following next step did you take the last time you had an asymptomatic patient with an elevated PSA level?[2]				
Recommended prostate biopsy	4	13%	5	16%
Repeated the PSA test within a certain time interval	22	71%	23	72%
Other	0	0%	4	13%
Not applicable	3	10%	0	0%

[1]Of those who answered affirmatively to the previous question

[2]Values are missing for three PCP respondents

© 2025 Lee JR et al. *JAMA Network Open*.

eTable 2. Demographics of Survey Respondents and Interview Participants

	Primary Care Providers (n=31)		*Urologists (n=32)*		*Interview Participants (n=29)*	
	**n**	**%**	**n**	**%**	**n**	**%**
Sex assigned at birth						
Male	17	55%	23	72%	29	100%
Female	14	45%	9	28%	n/a	n/a
^Age						
≤30	0	0%	1	3%	0	0%
30-39	5	16%	7	22%	2	7%
40-49	8	26%	10	31%	3	10%
50-59	10	32%	5	16%	9	31%
60-69	4	13%	7	22%	12	41%
≥ 70	4	13%	2	6%	2	7%
Ethnicity						
Hispanic or Latinx	2	6%	2	6%	n/a	n/a
Not Hispanic or Latinx	29	94%	30	94%	n/a	n/a
****Race***						
White/Caucasian	28	90%	24	75%	0	0%
Black or African American	1	3%	1	3%	29	100%
American Indian or Alaska Native	0	0%	1	3%	0	0%
Asian	4	13%	6	19%	0	0%
Native Hawaiian or other Pacific Islander	0	0%	0	0%	0	0%
Multiracial	0	0%	2	6%	0	0%
Other	0	0%	0	0%	0	0%
*****PSA/Prostate Cancer History***						
Received PSA testing	n/a	n/a	n/a	n/a	21	72%
Diagnosed with prostate cancer	n/a	n/a	n/a	n/a	14	48%
Never diagnosed with prostate cancer	n/a	n/a	n/a	n/a	15	52%
*****Degree***						
MD	29	94%	31	97%	n/a	n/a
DO	2	6%	0	0%	n/a	n/a
PA	0	0%	1	3%	n/a	n/a

© 2025 Lee JR et al. *JAMA Network Open*.

NP	0	0%	0	0%	n/a	n/a
Other	0	0%	0	0%	n/a	n/a
******Work experience (since completion of residency or fellowship)***						
0-4 years	4	13%	10	31%	n/a	n/a
5-9 years	2	6%	1	3%	n/a	n/a
10-19 years	9	29%	10	31%	n/a	n/a
≥ 20 years	16	52%	11	34%	n/a	n/a
******Main work setting***						
Urban	25	81%	29	91%	n/a	n/a
Rural	4	13%	2	6%	n/a	n/a
Other	2	6%	1	3%	n/a	n/a
******Practice type***						
Private practice	6	19%	10	31%	n/a	n/a
Academic/teaching	17	55%	17	53%	n/a	n/a
Community health center	6	19%	2	6%	n/a	n/a
Other	2	6%	3	9%	n/a	n/a

^Data missing for one interview participant

***Respondents were able to select more than one option**

****Data not available/applicable for survey respondents**

*****Data not available/applicable for interview participants**

© 2025 Lee JR et al. *JAMA Network Open*.

eTable 3. Prostate Cancer Screening Recommendations

Guideline	Screening recommendations for Black Individuals
American Urological Association	"Clinicians should offer prostate cancer screening beginning at age 40 to 45 years for people at increased risk of developing prostate cancer based on the following factors: Black ancestry, germline mutations, strong family history of prostate cancer." [1]
American Cancer Society	"[D]iscussion about screening should take place…at age 45 for men at high risk of developing prostate cancer. This includes African American men and men who have a first-degree relative (father or brother) diagnosed with prostate cancer at an early age (younger than age 65)."[2]
U.S. Preventive Services Task Force	"Based on the available evidence, the USPSTF is not able to make a separate, specific recommendation on PSA-based screening for prostate cancer in African American men" [3]

[1] **https://www.auajournals.org/doi/10.1097/JU.0000000000003491**

[2] **https://www.cancer.org/cancer/types/prostate-cancer/detection-diagnosis-staging/acs-recommendations.html**

[3] **https://www.uspreventiveservicestaskforce.org/uspstf/recommendation/prostate-cancer-screening#fullrecommendationstart**

© 2025 Lee JR et al. *JAMA Network Open*.
