# Supplementary material for: Patient and Physician Perceptions of Prostate-Specific Antigen Testing Among Black Individuals
Source: JAMA Netw Open. 2025 Sep 8;8(9):e2530946. doi: 10.1001/jamanetworkopen.2025.30946 (PMC12418120; doi:10.1001/jamanetworkopen.2025.30946)
Supplement: Supplement 2. — Data Sharing Statement [file jamanetwopen-e2530946-s002.pdf]

Data Sharing Statement

Lee. Patient and Physician Perceptions of Prostate-Specific Antigen Testing Among Black Individuals. *JAMA Netw Open*. Published September 08, 2025. doi:10.1001/jamanetworkopen.2025.30946

Data
Data available: No

Additional Information
Explanation for why data not available: We are unable to make interview data available to others due to confidentiality and privacy considerations.
